# Study on the Mechanism of Burr Formation by Simulation and Experiment in Ultrasonic Vibration-Assisted Micromilling

**DOI:** 10.3390/mi14030625

**Published:** 2023-03-09

**Authors:** Yuanbin Zhang, Zhonghang Yuan, Bin Fang, Liying Gao, Zhiyuan Chen, Guosheng Su

**Affiliations:** 1 School of Mechanical Engineering, Qilu University of Technology (Shandong Academy of Sciences), Jinan 250353, China; 2 Shandong Institute of Mechanical Design and Research, Jinan 250353, China; 3 School of Mechanical Engineering, Hefei University of Technology, Hefei 230009, China

**Keywords:** traditional micromilling, simulation, burr, ultrasonic vibration-assisted micromilling, size effect, cutting performance

## Abstract

Due to the strong plasticity of Inconel 718 and the significant size effect of micromachining, a large number of burrs will be produced in traditional processing. The addition of ultrasonic vibration during machining can reduce the burr problem. The mechanism of burr generation in traditional micromilling (TMM) and ultrasonic vibration-assisted micromilling (UVAMM) was analyzed by simulation, and verified by corresponding experiments. It is found that applying high-frequency ultrasonic vibration in the milling feed direction can reduce cutting temperature and cutting force, improve chip breaking ability, and reduce burr formation. When the cutting thickness will reach the minimum cutting thickness hmin, the chip will start to form. When *A*/*ƒ*_z_ > 1/2, the tracks of the two tool heads start to cut, and the chips are not continuous. Some of the best burr suppression effects were achieved under conditions of low cutting speed (*V*_c_), feed per tooth (*ƒ*_z_), and large amplitude (*A*). When *A* is 6 μm, the size and quantity of burr is the smallest. When *ƒ*_z_ reaches 6 μm, large continuous burrs appear at the top of the groove. The experimental results further confirm the accuracy of the simulation results and provide parameter reference.

## 1. Introduction

Nickel base alloys are widely used in modern aerospace engines because of their excellent high temperature properties [1]. Engine parts have complex structure and high machining accuracy. Micromilling is an important processing method, with high material removal rate and excellent processing flexibility, which can be used to manufacture precision parts [2,3], usually with a diameter of 1–1000 μm tool [4], which is also a common finishing method for nickel base alloys. However, it usually produces a lot of burrs when it is processed. Burr is an important machining defect that affects the quality of machined surface, will affect the performance of parts, and even lead to parts scrapping. In industrial production, deburring is tedious work [5], and the removal effect is often not ideal. Therefore, it is necessary to reduce the formation of burrs during machining. Scholars conducted various studies on the formation of burrs. Hashimura et al. [6] classified burrs into top burrs, inlet burrs, outlet burrs, inlet-side burrs, and outlet-side burrs according to their positions. Under normal conditions, the burr size can be reduced by changing processing parameters, such as feed speed, spindle speed, cutting depth, etc. [7,8,9,10]. The conical degree of the tool and the wear of the tool itself can also affect the size of the burr [11,12]. However, the effect is often not ideal. Furthermore, some researchers adopted the means of adding auxiliary technology to reduce the formation of burrs. Additional ultrasonic vibration during cutting can reduce the size of burrs [13,14]. Miranda Giraldo et al. [15] conducted milling research using biopolymers and found that when eddy current cooling is used, the growth of burrs can be effectively suppressed and the width of burrs can be significantly reduced. In order to further explore the mechanism of burr formation, many researchers simulated and analyzed the burr formation process. Sreenivasulu et al. [16] established a mathematical model for burr formation during drilling, and verified the feasibility of the model by combining it with experiments. Meng et al. [17] studied the cutting performance of carbon fiber reinforced polymer (CFRP) through finite element simulation, and found that the outermost fiber was not cut, but pushed to the side by the cutting edge, and then the fiber rebounded to form burrs. Yadav et al. [18] conducted a finite element simulation study on the formation of burr at the up-milling side of high-speed micromilling Ti6A14V. It was found that when the speed is increased from 10,000 r/min to 200,000 r/min, the burr size decreases sharply. Zou et al. [19] established a finite element model and found that the formation of negative burrs includes eight stages, in which the formation of negative shear bands and stress changes are the main reasons for the formation of negative burrs. The thicker the backup material, the smaller the burr size. Asad [20] used the finite element method to simulate different combinations of *V_c_*, feed speed, and tool edge geometry. It is found that the larger the tool tip radius is, the larger the outlet burr is. Srenivasulu et al. [21] developed a drilling model for aluminum 6061 and 7075 alloys, and found that temperature has no significant effect on the burr height. An et al. [22] proposed a new method for ultra precision cutting and deburring with special single crystal diamond tools, and carried out finite element simulation research, which proved that it is feasible. Chen et al. [23] found that applying vibration in the feed direction can inhibit the formation of burrs. The burrs at the top of the milling side are significantly reduced, and the average height is reduced by 87%. Chen et al. [24] found that microchannel with good surface quality can be obtained by machining with high spindle speed, small cutting depth and medium feed speed close to the tool cutting edge radius. Chen et al. [25] found that when the thickness to be cut is greater than the minimum cutting thickness, the cutting state can be changed to reduce the cutting force, tool wear, and the formation of burrs. In the cutting experiment, it is found that the burr width of ultrasonic vibration-assisted milling decreases from 25 μm to 5 μm compared with traditional milling. Le et al. [26] proposed a method for predicting side burrs and exit fractures of prisms. The results show that ultrasonic vibration can change the machining state and affect the formation of burr, and the number and size of burr can be greatly reduced by applying an appropriate amplitude. Wu et al. [27] established a finite element model and found that the outlet surface angle had an impact on burrs, and burr-free cutting could be achieved when the outlet surface angle was less than 45°. Efstathiou et al. [28] established a drilling finite element model, which can predict the best parameters to suppress burr generation. However, the research work on ultrasonic vibration-assisted micromilling processing parameters and the influence of vibration parameters on the burr size is still less, especially the research on micromilling Inconel 718 material is less, and research and analysis are needed to guide the micromilling work.

In this work, ultrasonic vibration-assisted micromilling was used to process Inconel 718, which restrained the generation of burrs. The tool tip trajectory during machining is simulated and the chip breaking mechanism is analyzed. For clarifying the role of ultrasonic vibration in the micromachining process, burr formation is simulated under different processing parameters. At the same time, traditional micromilling and ultrasonic vibration-assisted micromilling were used to machine high-temperature nickel base alloy for comparative analysis. Finally, the mechanism of burr formation under UVAMM is analyzed based on the simulation and experimental results.

## 2. Simulation and Experiment

### 2.1. Simulation Method

**Figure 1:**
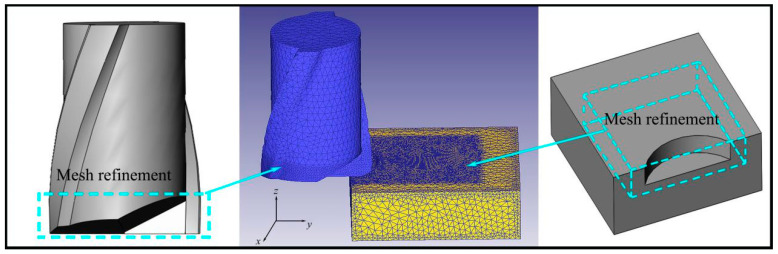
Simulation model and meshing.

The size and cause of the top burr are simulated with Deform-3D software (version number:V6.1; creator: SFTC; sourced from Key Laboratory for High Strength Lightweight Metallic Materials of Shandong Province). In order to improve the calculation efficiency and ensure the calculation accuracy, the tool and workpiece size are reduced in the same proportion, and the original geometric characteristics of the tool are retained. Table 1 shows the geometric parameters of the tool used, and Table 2 shows the simulation parameters of micromilling. The workpiece size is 0.3 mm × 0.3 mm × 0.1 mm, and the material is Inconel 718. According to the size of the tool and the workpiece, the geometric model of the tool and the workpiece is established in the SolidWorks software (version number: SOLIDWORKS EDU Edition 2016-2017-STAND-ALONE; creator: Dassault Systemes; sourced from Yantai Univ.), and the established model is imported into the software Deform-3D. The simulation process did not consider the influence of tool wear, so the tool model was set as a rigid body and the workpiece was set as an elastic–plastic deformation body. The local mesh refinement method is used to process the tool tip part and the uncut area of the workpiece. This simulation uses tetrahedral mesh to simplify the model and adapt to the boundary features in the mesh. The number of tool meshes is 30,000, and the number of workpiece meshes is 160,000. The model and mesh division are shown in Figure 1.

In order to better reflect the effects of strain hardening, strain rate strengthening, and thermal softening during the processing of high-temperature nickel-based alloys. In the Deform-3D modeling process, when defining the parameters of the material Inconel 718, the J-C constitutive model is used to simulate its plastic behavior, as shown in Equation (1), where *A* = 450 MPa, *B* = 1700 MPa, *n* = 0.6, *C* = 0.017, and *m* = 1.3 [29].

(1) $$\sigma =\left[A+B\varepsilon^{n}\right]\left[1+Cln\left(\frac{\dot{\varepsilon }}{\dot{\varepsilon_{0}}}\right)\right]\left[1-{\left(\frac{T-T_{room}}{T_{melt}-T_{room}}\right)}^{m}\right]$$

where σ is used to indicate the equivalent stress; ε is used to indicate the equivalent plastic strain;$\;\dot{\varepsilon \;}$ is used to indicate the equivalent plastic strain rate; $\dot{\varepsilon_{0\;}}$ is used to indicate the reference strain rate; *T* is used to indicate the temperature of the workpiece; *T_room_* is used to indicate room temperature; and *T_melt_* is used to indicate the melting temperature of the workpiece.

### 2.2. Experimental Method

**Figure 2:**
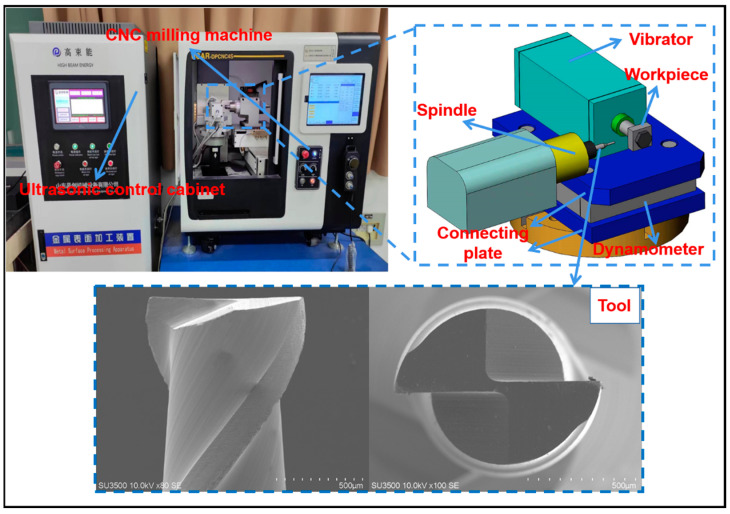
Experimental device.

The test machine and cemented carbide double edge end milling are shown in Figure 2. The burrs produced by micromilling grooves were studied by scanning electron microscopy (SEM).

The profile geometric parameters of micromilling tools are shown in Table 3. The cutting edge radius of the tool is 5 μm. The chemical element composition of workpiece material is shown in Table 4. The machining parameters are shown in Table 5, which is in accordance with Table 2 in order to compare with the simulation results. With *V*_c_ and ƒ_z_ as variables, two groups of single-factor experiments corresponding to the simulation were designed to study the influence of them on the size and shape of burrs generated by ultrasonic vibration-assisted micromilling.

## 3. Results and Discussion

### 3.1. Chip Formation

**Figure 3:**
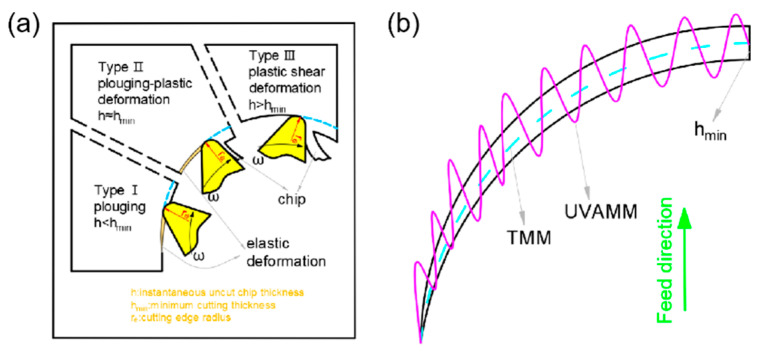
(**a**) Micromilling chip formation process in a single cutting cycle; (**b**) ultrasonic vibration increases h in cutting process.

Incomplete chip forming and fracture will eventually lead to burrs in micromilling. In a cutting cycle, the cutting thickness of the up-milling side will gradually increase, and the minimum cutting thickness will not be reached at the beginning. The material will only undergo elastic deformation without chip generation. With the continuous accumulation of cutting materials, the cutting thickness will reach the minimum cutting thickness h_min_. The stress and strain of the material will increase, and the chip will begin to form and still be an incomplete rebound of the material. Finally, when the stress in the cutting area reaches the yield limit with the increase in the cutting thickness, the material will mainly undergo plastic deformation. From the micro level, the lattice of the extruded cutting area will slip, and when the stress exceeds the fracture limit, the chip is formed normally. On the contrary, in a cutting cycle, the cutting thickness of the down-milling side will gradually decrease, and when the cutting thickness reaches a certain limit, chip breaking will occur, and some will become burrs. Therefore, the extrusion deformation process on the up-milling side will lead to burrs, and the incomplete formation of chips on the down-milling side will also lead to burrs. However, when ultrasonic vibration is introduced, even if the h at any time in TMM is less than h_min_, it will make h larger, h will periodically exceed him, and will change the cutting form. Figure 3 shows the three stages of chip formation, and the minimum cutting thickness is constant with the effect of ultrasonic vibration on cutting thickness.

### 3.2. Trajectory Equation of Cutting Edge

**Figure 4:**
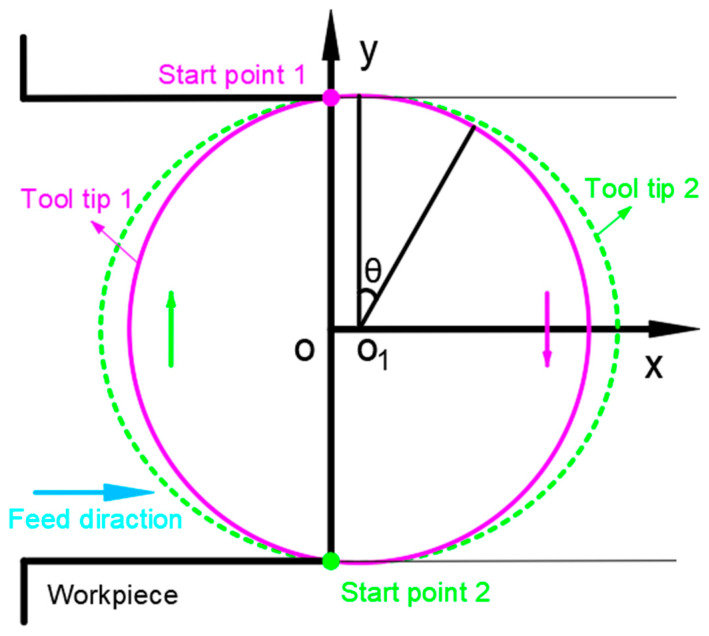
Schematic diagram of tool tip path.

During milling, applying high-frequency vibration along the tool feed direction will change the tool tip path, the chip formation process will also change, and will affect the generation of burrs. Therefore, it is necessary to analyze the running track of the tool tip. Figure 4 shows the trajectory of two tool tips during TMM tool tip movement. The solid line is the path of tool tip 1, and the dotted line is the path of tool tip 2.

In the coordinate system shown in Figure 4, the motion of the tool tip is decomposed into feed motion and rotation motion, and Equation (2) is used to represent the motion of two tool tips in TMM.

(2) $$\{\begin{array}{c} x_{i}=v_{f}t+{\left(-1\right)}^{i-1}rsin\left(\theta \right)\\ y_{i}={\left(-1\right)}^{i-1}rcos\left(\theta \right) \end{array}$$

In the experiment, the vibration signal along the tool feed direction is directly applied to the workpiece. In order to facilitate the synthesis of the motion path, the sinusoidal vibration signal applied to the workpiece is equivalent to the tool. Equations (3) and (4) represent the motion of two tool tips in UVAMM.

(3) $$\{\begin{array}{c} x_{i}=v_{f}t+{\left(-1\right)}^{i-1}rsin\left(\theta \right)+Asin\left(\lambda \omega t\right)\\ y_{i}={\left(-1\right)}^{i-1}rcos\left(\theta \right) \end{array}$$

(4) θ=2πnt

where $v_{f}$ is used to indicate the milling feed rate; *r* is used to indicate the tool radius; *n* is used to indicate the spindle speed; ω is used to indicate the ultrasonic signal angular frequency; *i* (*i* = 1,2) is used to indicate the *i*th tool tip; and *λ* is used to indicate the ratio of ultrasonic vibration signal frequency to spindle rotation frequency.

### 3.3. Simulation and Experiment Results

#### 3.3.1. Trajectory of Cutting Edge

**Figure 5:**
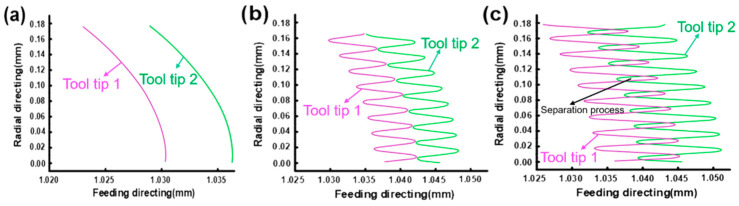
Simulation of tool tip trajectory (**a**) ƒ_z_ = 6 μm/z, *A* = 0 μm; (**b**) ƒ_z_ = 6 μm/z, *A* = 3 μm; and (**c**) ƒ_z_ = 6 μm/z, *A* = 6 μm.

The tool tip trajectory model can be used to predict chip formation. Figure 5 shows the influence of ultrasonic amplitude and ƒ_z_ on the two tool tip trajectories. When *A* is 0 μm, it is traditional milling, and the motion tracks of the two tool tips do not intersect, indicating that a cutting edge is continuous cutting in a cutting cycle. When A increases to 3 μm, the running track of tool tip 2 starts to be tangent to the running track of tool tip 1, indicating that a cutting edge starts to cut intermittently in a cutting cycle, and the chip is potentially broken. When *A* continues to grow, the running track of tool tip 2 intersects with that of tool tip 1. The intersection indicates that tool tip 2 will be separated from the workpiece at this moment, and the chip is not continuous. It shows that the chip breaking form can be changed by changing the size of *A* when ƒ_z_ is fixed. According to the motion track characteristics of ultrasonic vibration tool tips, when *A*/ƒ_z_ > 1/2, the tracks of two tool tips start to cut, and the chips are not continuous.

#### 3.3.2. Cutting Force and Cutting Temperature

Cutting force and cutting temperature can intuitively reflect the cutting process. In order to completely understand the processing characteristics of TMM and UVAMM, the cutting force and cutting temperature during the cutting process are simulated and analyzed.

**Figure 6:**
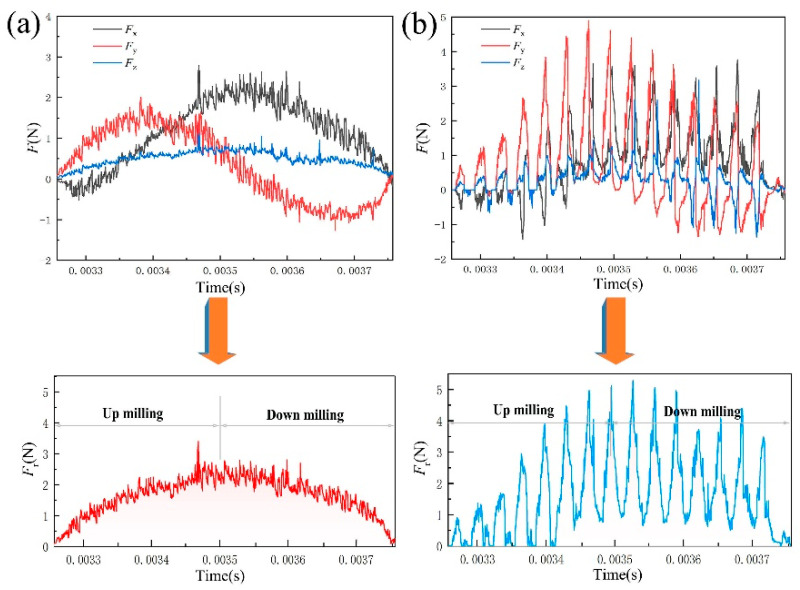
Cutting force changes in a single cutting cycle (**a**) TMM; (**b**) UVAMM.

Figure 6 shows the cutting force characteristics of TMM and UVAMM. The axial force (*F*_z_) is the driving force that pushes the chips to bend upward. The feed perpendicular force (*F*_x_) and the feeding force (*F*_y_) are the driving forces that promote chip growth. The resultant cutting force (*F*_r_) is calculated by Equation (5) and the curve is drawn. Figure 6 shows that, compared with TMM, when the tool suddenly contacts the workpiece after the vibration is applied, the cutting force quickly reaches the peak value, which is the contact impact between the tool and the workpiece, and then the cutting force decreases sharply at a high frequency, indicating that the cutting process of UVAMM is intermittent. Therefore, the plough effect on the up-milling side is weakened, and the extrusion deformation of the material is also weakened. On the down-milling side, it can be found that the cutting force dropped sharply, indicating that the chips were broken in advance, so no large burr will be formed on the top of the groove. The high frequency fluctuation of cutting force of UUVAMM results in the average value of the cutting force being lower than that of TMM.

(5) $$F_{r}=\sqrt{{\left(F_{x}\right)}^{2}+{\left(F_{Y}\right)}^{2}+{\left(F_{z}\right)}^{2}}$$

**Figure 7:**
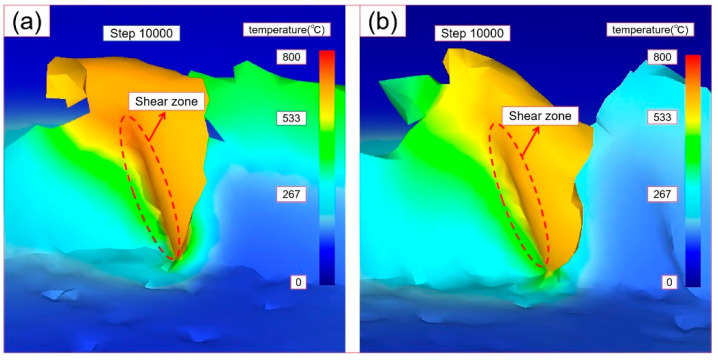
Distribution of cutting temperature field (**a**) TMM (*V*_c_ = 37.7 m/min; *ƒ*_z_ = 6 μm/z; *A* = 0 μm); (**b**) UVAMM (*V*_c_ = 37.7 m/min*; ƒ*_z_ = 6 μm/z; *A* = 6 μm).

Figure 7 shows the distribution of the cutting temperature field of TMM and UVAMM. It can be found from the temperature field that the highest temperature is mainly distributed in the shear deformation area and gradually diffuses to other parts of the workpiece. The temperature of the contact zone between the tool tip and the material is the highest, and the maximum temperature of TMM and UVAMM is 753 °C and 685 °C, respectively. At the same time, the temperature diffusion area of TMM is larger than that of UVAMM. The reason is that although the instantaneous cutting thickness of UVAMM is sometimes greater than TMM, because UVAMM is intermittent cutting, it is not conducive to the accumulation of cutting heat, reduces the heat generated by friction between the tool tip and the material, and provides more sufficient time for the heat dissipation process. Wang et al. [30] also pointed out that additional ultrasonic vibration can effectively increase the diffusion time and diffusion space of cutting temperature.

#### 3.3.3. Equivalent Strain Analysis

**Figure 8:**
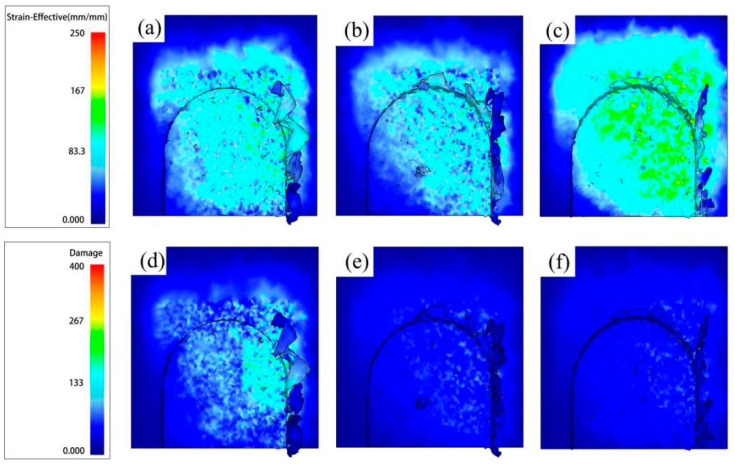
Equivalent plastic strain cloud image and damage cloud image with different *A.* (**a**,**d**) *A* = 0 μm; (**b**,**e**) *A* = 3 μm; and (**c**,**f**) *A* = 6 μm.

Figure 8 shows the distribution of equivalent plastic strain and damage in the cutting area when *ƒ*_z_ = 8 μm/z. It can be concluded from Figure 8a–c that the equivalent plastic strain is mainly distributed on the side where the chips are generated. The application of ultrasonic vibration expands the equivalent plastic strain range and increases the maximum equivalent plastic strain, which indicates that ultrasonic vibration will increase material deformation during the cutting process. In Figure 8d–f, as the *A* increases, the damage range and the maximum damage value decrease. The UVAMM is beneficial to remove materials. The material failure mechanism of UVAMM can be considered as the instantaneous plastic failure of material caused by high-speed impact load. Therefore, UVAMM is helpful to remove chips and reduce the residual burrs on the top of the milling groove.

#### 3.3.4. Burr Formation

**Figure 9:**
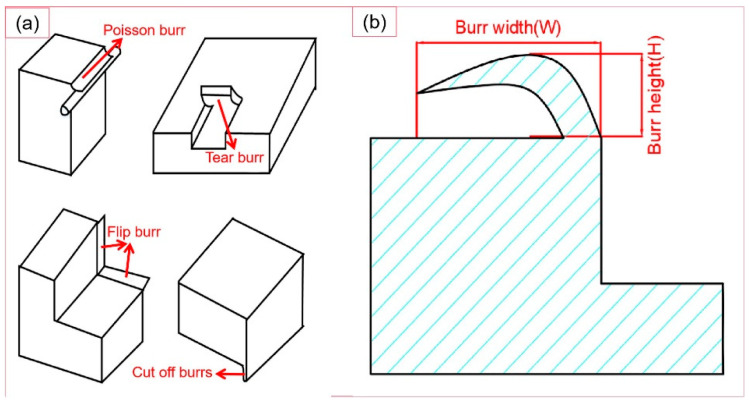
(**a**) Burr types; (**b**) burr size.

Burrs can generally be classified into four types, as shown in the Figure 9a. The size of burrs is usually defined in the way shown in the Figure 9b. This paper mainly compares the width of burrs.

(1) Burr formation process.

**Figure 10:**
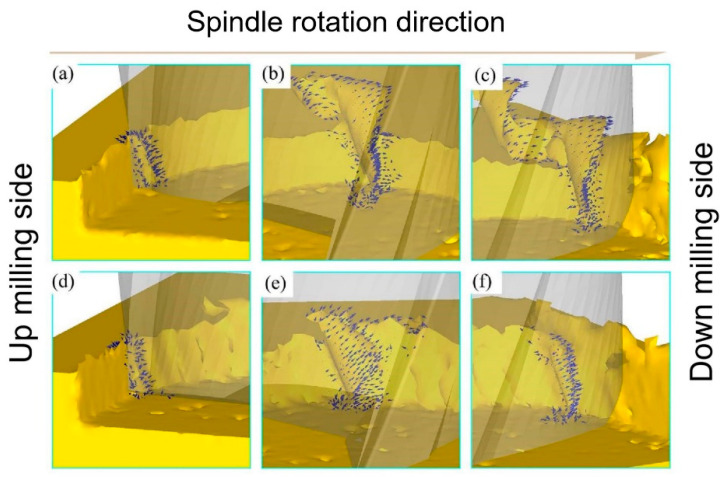
Simulation of burr growth (**a**–**c**) TMM; (**d**–**f**) UVAMM.

**Figure 11:**
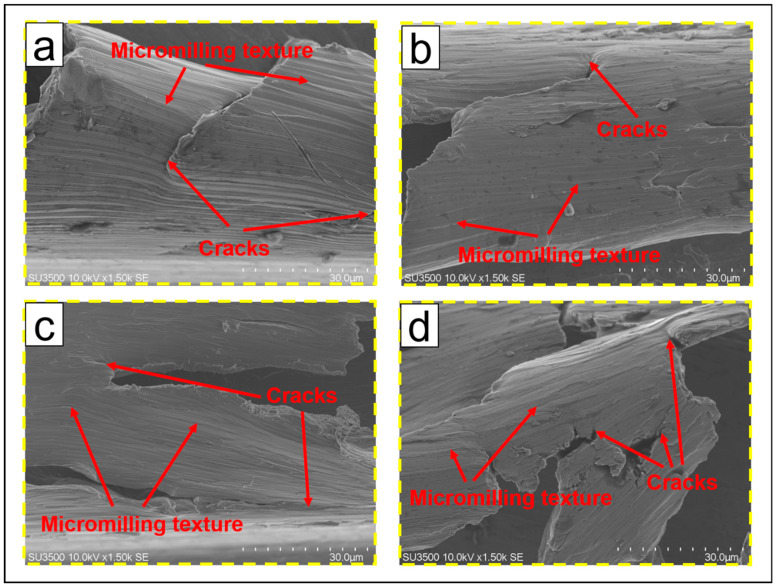
Burr morphology (**a**,**b**) TMM; (**c**,**d**) UVAMM.

Figure 10 shows the burr formation process of TMM and UVAMM. When the tool tip just touches the material, the size effect is particularly significant due to the large ratio of the cutting edge radius to the cutting thickness. In the TMM process, the part of the material continuously accumulates along the rotation direction under the extrusion of the tool tip, and the other part of the material is turned out of the groove and forms burrs on the top of the groove, as shown in Figure 10a. When the material accumulates to a certain value, a larger spiral chip forms, as shown in Figure 10b. The spiral chips continue to grow and form larger burrs on the down-milling side, as shown in Figure 10c. After that, due to the effect of high-frequency ultrasonic vibration, the extrusion of the tool tip on the material is weakened, the burrs on the up-milling side are reduced, as shown in Figure 10d. In addition, UVAMM enhances the destructive effect of the tool on the material. This is in accordance with the results of Figure 8. It can be found from Figure 11e–f that the chips of the up-milling side are smaller than that of the TMM, and the burr is serrated. With the chip growth, the chip size increases gradually. Due to the chip breaking phenomenon in the intermediate process, the chip is a C-type chip, and finally a small burr is formed on the down-milling side. Therefore, compared with TMM, UVAMM can significantly inhibit the growth of burrs and reduce the size of burrs. As shown in Figure 10a,b, the burr surface has a wide texture during TMM, which is formed by material accumulation during processing. After ultrasonic vibration is applied, larger flake burrs gradually change into smaller tear burrs and flocculent burrs, and the burr surface has a more dense texture. Because high-frequency ultrasonic vibration promotes crack growth, and dense vibration cracks remain on the burr surface. Ultrasonic vibration makes materials more vulnerable to damage and cracks more obvious, which is conducive to the transition from ductile fracture to brittle fracture, so the burr is more broken, and the burr size will be smaller, as shown in Figure 11c,d. Chen et al. [25] also found that UVAMM contributed to crack generation and material damage.

(2) Effect of ultrasonic vibration on burr formation at different *V*_c_.

**Figure 12:**
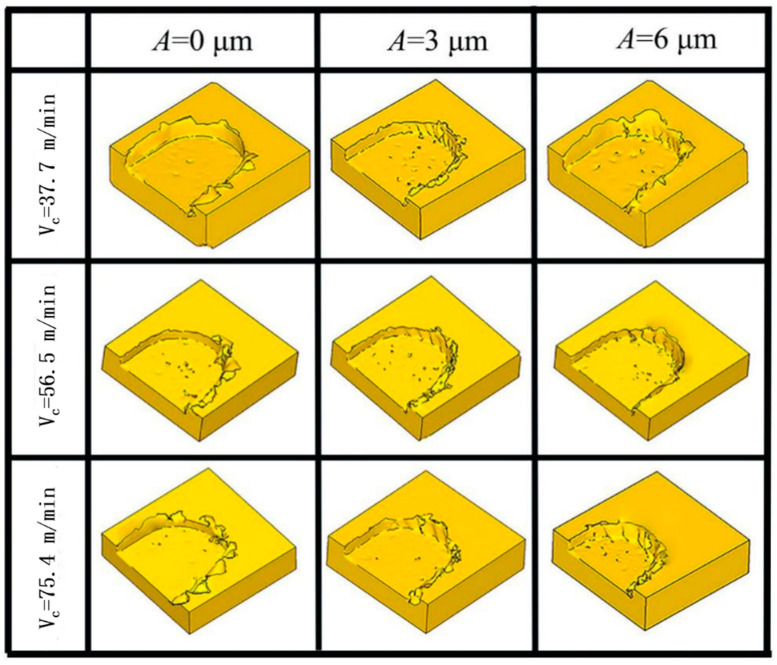
Simulation of burr morphology under different *V*_c_.

**Figure 13:**
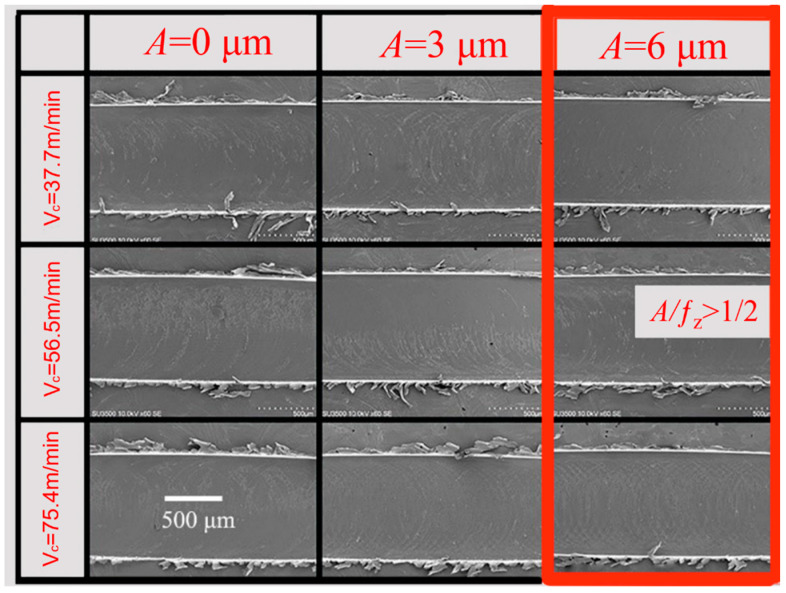
Morphology of burr under different *V*_c_ (SEM) (*f_z_* = 6 μm/z, *a_p_* = 50 μm).

Figure 12 shows the burr morphology generated by simulating TMM and UVAMM with different *V*_c_ (*f_z_* = 6 μm/z, *a*_p_ = 50 μm). It can be seen from Figure 12 that when the *V*_c_ is 37.7 m/min, large flake burrs are produced on the top of the groove. With the increasing of the *V*_c_, the burr size first decreases and then increases without ultrasonic vibration. When vibration is applied, the flake burrs gradually change to tearing burrs, and the size and quantity of burrs reduce. When the *A* is 6 μm, the size and quantity of burrs are the smallest. In addition, ultrasonic vibration has the most obvious effect on milling at medium and low cutting speed. Zhang et al. [31] also pointed out that the burrs become more with the increase in the cutting speed. Similarly, as shown in Figure 13, it was also observed in the above phenomenon in this experiment (in the Figure, in each groove, the down-milling side is located at the upper side). When traditional micromilling is carried out at *V*_c_ = 37.7 m/min cutting speed, a large number of complete sheet burrs are generated at the down-milling side and a large number of strip burrs are generated at the up-milling side. When *V*_c_ is increased, it is found that the size of burr is gradually reduced. Because the high cutting speed leads to a shorter single milling cycle time and it is difficult to produce plastic deformation, the burr is smaller. However, higher *V*_c_ will also produce higher cutting heat on the down-milling side, and the material will become soft, which is not conducive to chip fracture and promotes the generation of burrs. During UVAMM processing, it can be found that the burrs on the up-milling side and the down-milling side become more finely broken, because ultrasonic vibration causes the cutting process to be interrupted and the chip formation process is also interrupted, which significantly reduces the burr size.

(3) Effect of ultrasonic vibration on burr formation at different *f_z_.*

**Figure 14:**
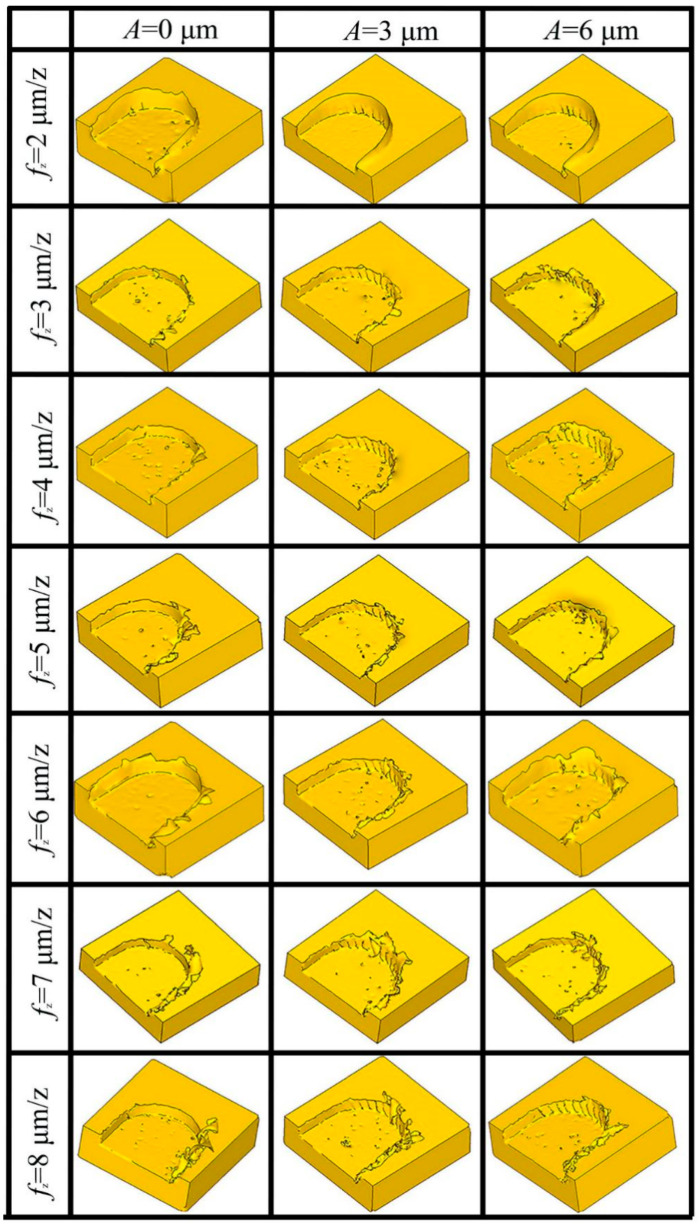
Simulation of burr morphology under different *ƒ*_z_.

**Figure 15:**
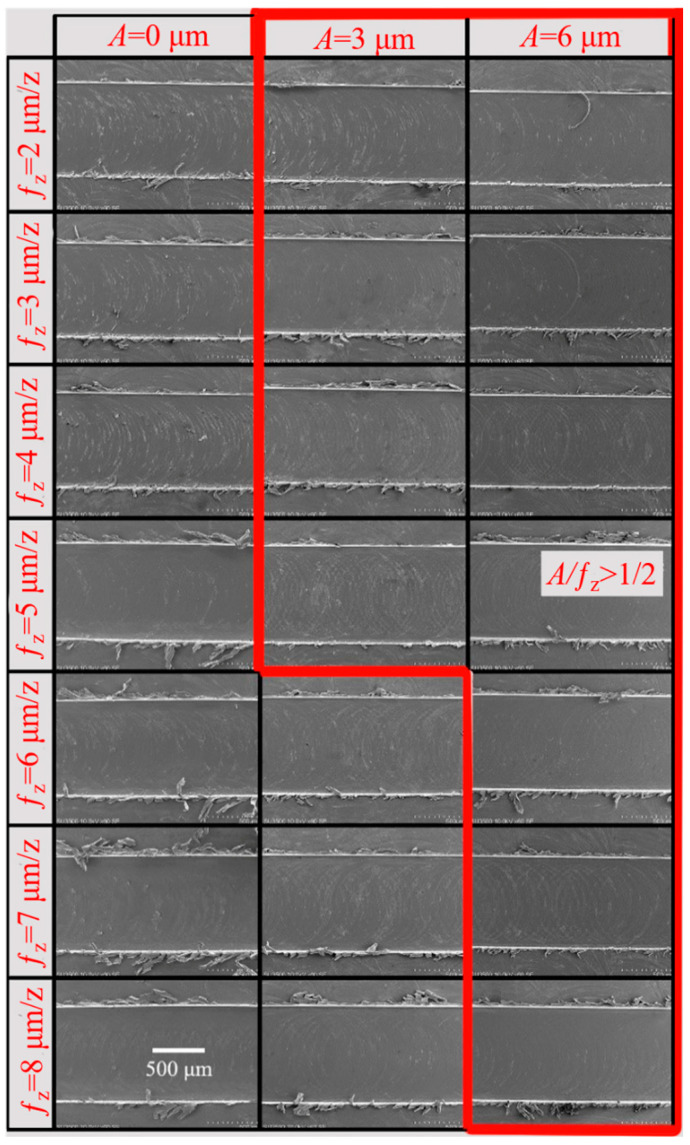
Morphology of burr under different *ƒ*_z_ (SEM); (*V*_c_ *=* 37.7 m/min, *a_p_* = 50 μm).

Figure 14 shows the burr morphology of a simulation by the two machining methods with different *f_z_* (*V*_c_ = 37.7 m/min, a_p_ = 50 μm). When the *f_z_* is 2 μm/z, the elastic deformation of the material is serious and chips are difficult to generate. The material is squeezed by the tool and slips upward, forming burrs. The material is easier to remove with the increase of *f_z_*. Large continuous burrs appeared on the top of the groove when *f_z_* reached 6 μm. Under the same *f_z_* condition, the burr generated by UVAMM was smaller than that by TMM, and the burr size decreased with the increase of A. This phenomenon is more obvious under large *f_z_* conditions, especially when *f_z_* is 5–7 μm/z and *A* is 6 μm. Similarly, as shown in Figure 15, it was also observed in the above phenomenon in the experiment (in the Figure, in each groove, the down-milling side is located at the upper side). When TMM is carried out, *f_z_* gradually becomes larger, resulting in an increase in the cutting amount, and finally leading to larger burr size. During UVAMM, due to the introduction of high-frequency vibration signals in the milling process, the instantaneous cutting thickness changed, which promotes the formation and separation of chips, and at the same time, the burrs are suppressed. This phenomenon is more obvious under the condition of large *f_z_*. When *f_z_* reaches 8 μm/z, the additional ultrasonic vibration cannot significantly suppress the burr, mainly because the *f_z_* is too large to meet the chip breaking condition. Larger chips do not break, they stay at the top of the groove and form burrs. When *f_z_* is small, ultrasonic vibration mainly reduces the size effect and changes the chip formation process, thereby inhibiting the formation of burrs. When *f_z_* is large, the burr size decreases because ultrasonic vibration changes the instantaneous cutting thickness in the milling process and improves the chip-broken ability.

## 4. Conclusions

The influence of UVAMM on cutting force, cutting temperature, and burr size was analyzed by simulation and experiment. The research findings are as follows:

(1) When ultrasonic vibration is applied and the ratio of *A* to *ƒ*_z_ reaches a certain limit, the UVAMM processing mode will change, and the cutting process will no longer be continuous, but will change to intermittent cutting;

(2) Compared with TMM, the average cutting force and cutting temperature of UVAMM are reduced, the strain range expands by applying ultrasonic vibration, and the energy required for damage reduces;

(3) Through simulation and experiment, it is found that the additional ultrasonic vibration can effectively reduce the burr size in the milling process. The bigger *A* is, the better the burr suppression effect is;

(4) UVAMM can reduce the size of burr by reducing the size effect and promoting the fracture of burr. When *A* is 6 μm, the size and quantity of burr is the smallest;

(5) When the *ƒ*_z_ is small, UVAMM can reduce the size effect and change the chip formation process to suppress the formation of burr. When *ƒ*_z_ is large, the burr size can be reduced by changing the instantaneous cutting thickness to improve the chip breaking ability.

## Figures and Tables

**Table 1:** Geometrical parameters of Simulation model.

Geometrical Characteristic	Tool Diameter (mm)	Blade Length (mm)	Cutting Edge Radius (μm)	Helical Angle (°)
Parameter value	0.2	4	5	36
Geometrical characteristic	Rake angle of side edge (°)	Rear angle of side edge (°)	First rear angle of base edge (°)	Second rear angle of base edge (°)
Parameter value	0	9	9	16

**Table 2:** Simulation parameters.

Experiment Number	Cutting Speed *v_c_* (m/min)	Cutting Depth *a*_p_ (μm)	Feed per Tooth *ƒ*_z_ (μm/z)	Amplitude *A* (μm)	Vibration Frequency *ƒ* (kHz)
Simulation 1	37.7 56.5 75.4	50	6	0 3 6	32
Simulation 2	37.7	50	2 3 4 5 6 7 8	0 3 6	32

**Table 3:** Geometrical parameters of micromilling tool.

Geometrical Characteristic	Tool Diameter (mm)	Blade Length (mm)	Cutting Edge Radius (μm)	Helical Angle (°)
Parameter value	1	4	5	36
Geometrical characteristic	Rake angle of side edge (°)	Rear angle of side edge (°)	First rear angle of base edge (°)	Second rear angle of base edge (°)
Parameter value	0	9	9	16

**Table 4:** Main chemical components of Inconel 718.

Element	Ni	Cr	Fe	Nb	S	Ti
wt%	55.54	18.77	18.17	4.78	1.67	1.07

**Table 5:** Machining parameters.

Experiment Number	Cutting Speed *v_c_* (m/min)	Cutting Depth *a*_p_ (μm)	Feed per Tooth *ƒ*_z_ (μm/z)	Amplitude *A* (μm)	Vibration Frequency *ƒ* (kHz)
Experiment 1	37.7 56.5 75.4	50	6	0 3 6	32
Experiment 2	37.7	50	2 3 4 5 6 7 8	0 3 6	32

## Data Availability

The data supporting this study’s findings are available from the corresponding author upon reasonable request.
